# Individual Mental Abiities vs. the World’s Problems

**DOI:** 10.3390/jintelligence6020023

**Published:** 2018-04-16

**Authors:** Jonathan Baron

**Affiliations:** Department of Psychology, University of Pennsylvania, 3720 Walnut St., Philadelphia, PA 19104, USA; baron@upenn.edu

**Keywords:** Flynn effect, actively open-minded thinking, cognitive style, political judgment

## Abstract

The major problems in the world today are problems of government or the lack of it. Thus, the relevant parts of intelligence are those that make for good citizenship, such as supporting the best candidates and policies. I argue that dispositions, as well as capacities, are part of intelligence, and that some dispositions are the ones most crucial for citizenship, particularly the disposition to engage in actively open-minded thinking (AOT) and to apply it as a standard for the evaluation of the qualifications of authorities and leaders. AOT is a general prescriptive theory that applies to all thinking. It affects the aptness of conclusions and the accuracy of confidence judgments, and it reduces overconfidence when extreme confidence is not warranted. AOT may be affected by different factors from those that affect other components of intelligence and thus may undergo different changes over time. Whatever has happened in the past, we need more of it now.

## 1. Introduction

The question of this Special Issue is “...why are there so many unresolved and dramatic problems in the world, and what can be done about it?” In particular, what is the role of cognitive abilities in resolving these problems.

As I see them, most of the big ones can be described as failures of government. Here is a rough classification:

1. In many poor countries, government simply does not function [1]. In the so-called Democratic Republic of the Congo, government cannot prevent the rise of a feudalism of gangs and tribes, all heavily armed. And what is left of the government is hopelessly corrupt. Often, in cases such as Venezuela, situations like this have arisen from support for a bad government that took over from a relatively benign one.
2. In less-poor countries, and even some relatively rich ones, the function of government is hampered by widespread corruption, “crony capitalism”, and the social norms that support these practices [2,3]. Payola becomes a way of life, and it does not seem so bad to accept it when so many other are doing the same. The system maintains itself as a vicious circle.
3. In other countries, including some of the richest democracies along with others, voters accept unsupportable theories about the nature of their problems (e.g. [4]), leading to the adoption of policies that oppose the well-being of the citizens who supported them.
4. Some of these policies are isolationist: opposition to trade and immigration, unwillingness to participate in international agreements, and so on. These also threaten to create another vicious circle in which nations respond to each other in kind, thus losing the benefits of many kinds of international cooperation. And these policies often cause great harm to outsiders (such as potential refugees), who are simply left out of the moral calculus of the citizens who are in control, and, hence, their government ([5], especially ch. 6; [6], especially ch. 4).
5. At the opposite extreme of (1), world government is also not functioning. We have some international institutions, but they are weak and becoming weaker from increased isolationism of their members, and they do not coordinate with each other. This happens at a time when the human population, and its use of resources, have expanded to the point where their effects on the environment seriously threaten further increases in the world’s standard of living [7]. We are now depending on scientific, technological, and administrative advances not yet made, or fully known, in order to provide sufficient food, water, and energy even for the population we have [8].

Omitted from this list is one commonly invoked cause of problems, namely, the behavior of individuals outside of their role as citizens. It is sometimes said, for example, that a solution to global warming is for each of us to reduce our carbon footprint. There are two problems with this idea. First, it is difficult to do this without a lot of help from government, in the form of relevant infrastructure and changes in laws and regulations. Second, the capacity of people to sacrifice their own well-being for the good of others is limited. Of course, it is variable: some people are more willing than others to make sacrifices. But even those people need information about what sacrifices will matter. And it is doubtful that further exhortation from activists will increase the average level to the point where it makes a serious dent in the world’s problems. (The exhortation has been going on for a while.) It is not too much to expect people to recycle their trash (even in the absence of penalties for not doing it) or to reduce their water use during a drought. But the level of sacrifice required to prevent continued unsustainable draining of aquifers is much greater. It requires a combination of government coercion and government support of alternative water sources. Likewise, fishers are not going to reduce their catch to the point of sustainability of the fishery unless they are threatened with punishment for over-fishing ([6], ch. 1). We need government.

How can individuals help solve the problems of government? I think that the answer is different for different problems. In particular, the first problem, non-functioning national government, is not easily solved by individual citizens voting for politicians who will fix it. The “system” is too powerful. What is required is something more like revolution. That requires a few courageous and inspiring leaders, like Nelson Mandela, and a large number (although not necessarily a majority) who are willing to put their lives on the line for a cause [9]. The necessary traits thus do not have much to do with the sort of abilities measured by the IQ test, except perhaps in the leaders, and even for them these traits are not the critical ones. Still, after the revolution, the first problem can turn into the second (as has been a danger in South Africa).

In all but the last of these problems, the means for solving the problems are those of ordinary democracy.^1^ I have suggested [10] that, in order for a democracy to function well, its citizens need to endorse three social norms, which I called cosmopolitanism, anti-moralism, and actively open-minded thinking (AOT). The first two are specific to politics, so they are not of concern here, except that they may both be facilitated by AOT.^2^ The third is the main topic I shall address here, eventually. Note that AOT has many functions. It is an individual disposition, a social norm, and a standard for judgment.

First, a little history from a biased perspective, my own.

## 2. Dispositions, Abilities, and Intelligence

In 1985, I published *Rationality and Intelligence*, which argued that rational thinking was part of intelligence. This argument had two steps. The first was to define intelligence in a way that did justice to our use of the term in psychology but was not limited to “whatever the intelligence test measures” and not dependent on the assumption of a g factor (a concept often used to by-pass the need for a clear definition, since its existence seems to imply that “it doesn’t matter much how you measure it”). I concluded roughly that intelligence consisted of a bunch of potentially general traits (capacities and dispositions), each of which would help people achieve their rational goals, whatever these goals might be.

“General” meant that their definition was not confined to a particular type of mental task, or a particular subject matter. We could then ask the empirical questions of whether each trait was correlated across different tasks, and whether these different manifestations had common influences, such as transfer of learning in the case of dispositions, or biological substrates in the case of capacities.

Importantly, I saw no reason to exclude thinking dispositions such as perseverance oe open-mindedness. These fit the definition, and they surely influence essentially all manifestations of intelligence. Why should we regard them as artifacts? Measures of intelligence could try to exclude them, but do not. For example, we could attempt to measure pure capacities such as mental speed, or memory storage, using sophisticated cognitive tasks, but we do not do this except in experiments. Yet many writers today implicitly assume that intelligence is the biologically limited part and the contribution of acquired “personality” traits is an artifact (e.g., [11]). If they are separate, then what is intelligence?

Given that dispositions are included, which ones are relevant? To answer this question, we need a theory of rational thinking, defined now as that sort of thinking that helps people pursue their rational goals, whatever these goals might be.^3^ I then went on to state a theory of rational thinking, which has been modified a bit over the years, both in my own mind and in my publications (e.g., [12,13]).

The theory includes a simple framework for the description of all thinking, regardless of topic, and then applies standard normative models from decision theory to that framework. The result is a prescriptive model, AOT, which refers to objects available to the thinker, so that, in principle, it is meaningful for someone to try to follow the prescriptions in question. That is, it is not about the criteria of success and failure, but, rather, about the processes under the thinker’s control. The model does not require any additional domain-specific knowledge. Of course, good thinking is more effective when such knowledge is available.

AOT has a different purpose than that of other approaches to the analysis of thinking for the purpose of making thinking better. It concentrates narrowly on thinking itself, described in terms that are neutral with respect to content hence not specific to any particular type of thinking. The goal is more to find what is common to all forms of good thinking, and potentially to provide a unifying explanation of why they are useful. Other approaches (e.g., [14]) consider a broader concept, such as wisdom, and try to list all the traits and abilities that contribute to it. Some of these (e.g., tacit knowledge) have little to do with thinking in the more limited sense, and others (e.g., “good judgment”) seem to refer to criteria of success rather than prescriptions to be followed.^4^

The proposed framework analyzes thinking into search for various objects (described shortly) and inference from the resulting findings of the search. In ordinary thinking, these two steps are often interleaved through several episodes of search and inference. The objects consist of possibilities (answers to the question that inspired the thinking), evidence (objects that bear on the value of the possibilities), and goals (criteria that determine how each piece of evidence affects the value of each possibility). Search may be directed in various ways, but the main parameters of interest are its extent and its direction, with direction defined as for or against a currently favored possibility. The direction parameter may also be applied to inference.^5^

Rational thinking determines search and inference. Search is optimal so long as the benefit of additional search is still greater than its cost. Search is “fair” when it optimally distinguishes possibilities (possible answers to the question at hand). For example, it is wasteful to look for reasons why a favored option is best, if one is going to choose that option anyway. Inference depends heavily on the situation, but, again, in order to be fair it must not favor possibilities that are already strong.

Search should be sufficiently extensive for the task at hand. The optimal amount of search is determined by a balance between the cost of search and its potential benefit in terms of increased accuracy. If you know that additional search cannot lead to a change of mind, search is a waste of time. Search is most valuable when you are unsure, when the task is important, and when the prospect of finding something is good. More generally, the potential benefit of search depends on the expected-utility difference between the best possibility and the current favored possibility; this expectation, in turn, depends on the probability that new information will lead to a change in the correct direction (which is lower if the current possibility is more likely to be the best one). The cost of search depends on factors such as time pressure (high cost of delay) and whether the search is enjoyable or painful in its own right. At this point, with a few additional assumptions, the theory is clear enough to be programmed on a computer, although this has never been done.

An important aspect of good thinking, beyond accuracy in choosing the best possibility, is appropriate confidence. Reports of confidence should depend on the difference between the current value (strength) of the favored possibility and the total value of the alternatives. Confidence should depend on the thinking that has been done, or the lack of it. High confidence that one possibility is optimal is justified by extensive examination of the evidence for that possibility and others. (This does not have to be thinking of each citizen. As I shall point out, citizens rely heavily on others. But someone has to have done it.) Confidence should also depend on the balance of arguments for competing possibilities. When evidence is lopsided favoring one possibility, confidence in that one should be higher. Finally, confidence in politics (for example) should be low when little thinking has affected a particular political opinion. In many cases, extensive search is not worth the cost, and people rationally hold opinions that have not been subject to examination. But they should know that they are doing this and thus refrain from confidently accepting their tentative conclusions as justified, and they should refrain from imposing them on others. In the words of a bumper sticker: “Don’t believe everything you think.”

When we look at how people actually think, we find a few systematic and general departures from this model of rational thinking. Many of these departures are biases that favor conclusions (possibilities) that are already favored. People tend to search selectively for evidence that favors these possibilities, whether they search for external information [16] or internal information based on memory [12,17,18]. For example, Perkins et al. [17] asked students to write down their thoughts on issues that were “genuinely vexed and timely” and that could be discussed on the basis of knowledge that most people have, e.g., “Would providing more money for public schools significantly improve the quality of teaching and learning?” Most students gave more arguments on their favored side, “myside” thoughts, than on the other side. When the students were asked to try harder to think of arguments on each side, they thought of very few additional myside arguments but many additional otherside arguments. Left to their own devices, then, the students looked primarily for reasons to support their initial opinion, but out of biased search rather than lack of ability or knowledge.

People also use evidence in a way that supports their pet conclusions [19,20], even to the point of taking the same piece of evidence to favor different conclusions depending on which conclusion they favor [21]. These biases together are called “confirmation bias” or “myside bias” in the literature.^6^

In general, people search too little when search is warranted. We know this mainly from the fact that people who tend to search more do better in a variety of real-world manifestations of intelligence, such as school performance [22] and forecasting [23]. Yet the amount of search need not be correlated with fairness in the direction of search, nor with fairness of inferences, as I shall discuss.

Finally, confidence in judgments is generally too high when judgments are difficult. In most studies of the accuracy of confidence judgments, subjects are asked to provide answers to questions of fact plus a probability that their answer is correct. When the questions are difficult, the mean probability assigned by most subjects to a batch of questions is considerably higher than the proportion that they answer correctly [24]. Political issues, because they are often controversial, are difficult in the relevant sense. AOT reduces overconfidence, especially unjustified extreme confidence [18,25]. People who look for reasons against their favored option are likely to find them and reduce their confidence accordingly.

## 3. AOT and Politics

All aspects of intelligence are potentially relevant to citizenship of the sort that we need. Smarter people are more able to comprehend some of the issues that they need to understand in order to have informed opinions about policies, especially economic policies (e.g., [4,26]). They are also more likely to get the kind of education that will give them relevant background in many different fields. But AOT plays an outsized role among the aspects of intelligence relevant to politics.

### 3.1. The Benefits of AOT for Our Own Thinking

First, AOT helps individuals think through the issues on the table. Issues that citizens face tend to be ones with arguments for competing views, if only views about how to overcome the forces of inertia. Openness to arguments on different sides can make citizens more likely to change their mind in the direction of good arguments. Change need not be complete to be beneficial. A little doubt can be a good thing.

Nor does change need not be immediate. When we have thought about something long enough to have reduced confidence, we are more open to additional arguments. Lower confidence rationally increases the utility of additional information, so it is more likely to be sought. Reduced confidence also makes switching more likely when it is warranted by the evidence at hand.

Second, AOT permits better cooperation between political factions. Successful negotiation, in general, usually involves trade-offs on several attributes, such as working hours and salary in the case of labor negotiations [27]. Ideally, each party gives up on those attributes that is of greater concern to the other party. Such “log rolling” (or “integrative bargainins”) is more likely when the parties are aware of the weaknesses in their own original positions.

Similarly, AOT ought to reduce the polarization and fanaticism that often ties up political systems in knots. It is extremely unlikely that any political party or pressure group is absolutely right on every issue. Those who realize this are surely more willing to compromise.

### 3.2. Relation between AOT Norms and Actual Thinking

Baron [28,29] argued that beliefs affect what people do, and supported this with correlations between subjects’ beliefs about the nature of good thinking and the subjects’ own thinking. Stanovich and West [30,31] constructed a questionnaire that emphasized similar beliefs. Several papers (reviewed by [32] and [33], Table 1), found correlations between this belief scale and other tests, some of which measured biases described in the literature on judgment and decision making, including (but not limited to): Baserate Neglect, Conjunction Fallacy, Framing Effects, Anchoring Effect, Sample Size Awareness, Regression to the Mean, Temporal Discounting, Gambler’s Fallacy, Probability Matching, Overconfidence Effect, Outcome Bias, Ratio Bias, Ignoring P(D/H), Sunk Cost Effect, Risk/Benefit Confounding, Omission Bias, Expected Value Maximization, Hindsight Bias, Certainty Effect, Willingness to pay/Willingness to accept, and Proportion Dominance Effect.

Baron selected items from the Stanovich/West [30,31] scale and added others to make a short form more appropriate for the general population, designed to assess beliefs in particular. Example items are as follows: “People should take into consideration evidence that goes against their beliefs.” and “Changing your mind is a sign of weakness.” This short form has had considerable success in predicting the results of other tasks such as perceptual judgments and reduced over-confidence in them [34], accuracy in geo-political forecasting [23], utilitarian moral judgment, and problem solving ([35], using a slightly extended version).

The fact that this short belief measure (and others like it) correlates with task performance suggests that efforts to explain to people the value of AOT, thus changing their beliefs to make them more favorable toward AOT, could result in improved performance on many tasks that involve thinking. Gürçay-Morris [18] attempted to do this with a short training module, with some short-term success in reducing overconfidence.

### 3.3. AOT as a Set of Norms for Evaluation

AOT involves a set of thinking dispositions, but, in those who understand it, it also provides a set of norms (standards) for the evaluation of anyone’s thinking, including the thinking of others [36]. Indeed, John Stuart Mill was perhaps the clearest 19th century advocate of what I am calling AOT. In *On liberty* ([37], ch. 2), he writes (as part of a longer argument): “The whole strength and value, then, of human judgment, depending on the one property, that it can be set right when it is wrong, reliance can be placed on it only when the means of setting it right are kept constantly at hand. In the case of any person whose judgment is really deserving of confidence, how has it become so? Because he has kept his mind open to criticism of his opinions and conduct. Because it has been his practice to listen to all that could be said against him; to profit by as much of it as was just, and expound to himself, and upon occasion to others, the fallacy of what was fallacious.”

Individual citizens do not have the time or background to delve deeply into policies concerning trade, immigration, crime or almost anything [36]. Partly, this is a function of the low expected-value of spending time informing ourselves, but even if we are passionately involved, we cannot get to the bottom of all the issues we face. Too much is known for any one person to get to the bottom of almost anything. We must rely on the conclusions of others, and we must be able to distinguish relatively trustworthy sources from those that express gut-level intuitions as if they were proven facts or pearls of wisdom [38,39].

For example, science, and many other forms of scholarly inquiry (especially philosophy, these days), are based on actively open-minded thinking (AOT), refining themselves by challenging tentative beliefs. Astronomy differs from astrology because the latter has no standard procedures for thinking critically about its assertions. The same applies to a great deal of religious doctrine. Science, by contrast, engages in AOT at least as a group, if not within the heads of individual scientists. Scientists are rewarded (with publications, grants, promotions, jobs) for finding problems with the conclusions of other scientists. Individual scientists also try (perhaps not always hard enough) to anticipate possible criticisms before they try to publish something. These practices make science effective in approaching truth and understanding ever more closely.

Likewise, the application of the norms of scholarly inquiry, including AOT, in government itself can improve its effectiveness [40]. It would be helpful if citizens understood the value of these advances.

AOT is not just about “critical” thinking; as that term suggests a skeptical attitude, possibly leading critical thinkers to doubt even when they should trust. The understanding of AOT leads to trust insofar as trust is warranted, and this need not involve looking for flaws, as long as we know that others have done so on our behalf.

Populist, and often authoritarian, politicians gain power with the support of those who do not apply the norms of AOT to evaluate their thinking. These politicians are full of confident pronouncements of propositions that others find weak, subject to objections that are sometimes obvious with a little thought. But the air of confidence serves as a false signal of true expertise.

We have no better way. Alternatives such as “faith”, “the heart”, or acceptance of the word of authority have no built-in mechanism for self-correction. If their conclusions are wrong, we have no way to know, and, therefore, we also have no way to know when they are right.

## 4. The Flynn Effect and Possible Cultural Effects on AOT

The Flynn effect is a well-documented increase in IQ over several decades and several nations [41]. Recently, it may have hit a ceiling or even begun to decline in some countries (e.g., [42,43,44]). Of interest here is in which aspects of intelligence changed.

IQ tests are usually thought to measure fluid and crystallized intelligence. Crystallized intelligence is the result of accumulation of knowledge, hence a product of various capacities and environmental influences. Good learners will learn more words throughout their lives, so vocabulary is a reasonable crystallized measure of past learning ability, although of course it is affected by opportunity as well.

Fluid intelligence is usually thought to concern more direct measurement of biologically limited capacities such as mental speed. However, many of the measures of fluid intelligence involve problem solving, such as the Raven’s Progressive Matrices, which are also related to measures of cognitive style. For example, many problem-solving tasks show positive correlations between accuracy and response time (e.g., [26,45]). Such results must overcome any negative correlations resulting from any positive influence of mental speed on both accuracy and response *speed*.

All the measures I know that concern cognitive abilities or reflective cognitive style (including measures of AOT) correlate with each other, even when the latter are measured by questionnaires (e.g., [23,46]). Thus, the “positive manifold” that led Spearman to postulate the existence of a g factor seems to extend to dispositions as well as abilities. These correlations may exist for many reasons. High levels of basic capacities (such as mental speed, or durable memory storage) may be helpful in learning to think effectively, just as they help learn almost anything else. Or tests that attempt to measure such capacities might be less pure than they seem. Vocabulary items, for example, sometimes can be solved as problems when words are unknown, and even an apparent speed test such as the digit-symbol task can benefit from a thoughtful approach to choice of a strategy for doing the task. Or environmental influences may themselves be correlated, e.g., good nutrition, health care, and education (of the sort that encourages a more reflective cognitive style), as suggested by Pietschnig and Voracek [41]. Good thinking can even lead to better physical health.

These correlations, and their possible causes, make it difficult to distinguish effects, such as those of historical change, on capacities, acquired knowledge and dispositions. Differences in correlations may result from differences in reliability of measures (or validity in the sense of measuring what they are supposed to measure).

I could find no attempts to examine historical changes in cognitive style. All the evidence suggests that cognitive style is heavily influenced by culture. For example, it is correlated with aspects of religion, which is surely influenced by upbringing (e.g., [35,47]). It thus may be more sensitive to cultural change.

A possible candidate for examination of cultural change is the dimension of reflection/impulsivity (R/I) in problem solving. As argued by [35], this is a measure that can be defined in any problem-solving task. One way to do it is to measure the log of mean response time in the task, and the accuracy rate. (Use of the log makes the distribution closer to normal, thus removing what would otherwise be the excessive influence of long responses.) Then convert both to z scores and take the *sum*. (Note that the *difference* might be seen as a measure of raw mental power, which would consist of speed as well as accuracy.) At one end of the resulting continuum, reflective subjects would be slow and accurate. At the other end, impulsives would be fast and inaccurate. The measure thus assesses the willingness to trade off accuracy for speed (or speed for accuracy). It is quite general across tasks, so long as they do not involve time pressure. In many cases, the response-time measure correlates just as highly (positively) with other results as does the accuracy measure (the one usually used) [35]. Thus, much of the predictive power of problem solving tasks may be the result of the cognitive style of R/I.

One of the problem-solving measures that can be used for this purpose is the Raven’s Progessive Matrices. In its standard form, there is little or no time pressure. Yet I was unable to find any articles that reported response times, as well as accuracy. Someone must have relevant data possibly going back as far as 1936, but I could not find them. Such data could allow us to examine historical changes in R/I.^7^

Even if this could be done, though, it is not the best measure of AOT. I have argued (e.g., [29,35]) that reflective thought can be characterized on two dimensions, one of which is R/I, which concerns the tradeoff between the benefits and costs of additional search. The other is direction, specifically whether the thinking is biased toward possibilities that are already strong, both in search and inference, as distinct from being fair.^8^ AOT and R/I have different correlates. If we assume that the Cognitive Reflection Test (CRT) is primarily a measure of R/I (insofar as it is sensitive to general dispositions, as distinct from specific knowledge), then it is of interest that it has different correlates from questionnaire measures of AOT. For example, Baron [49] noted that AOT (the short scale) correlates 0.27 with self-reported political liberalism in a representative U.S. sample, while the CRT does not correlate at all in the same sample. Raven’s test might be much like the CRT.

AOT does not assume that more thinking is always better. AOT is primarily concerned with direction, and with appropriate confidence. The question of how much thinking is warranted is, as I argued earlier, part of a broader question concerned with the trade-off between amount and confidence. That said, some correlation between AOT and R/I is to be expected, if only because any attempt to be fair will require some search for counter-evidence as well as for evidence, where myside bias requires only the latter. And, even a long search for confirming evidence may turn up counter-evidence instead. Still, it would be nice if we had measures of direction as well as amount.

One possible way to study AOT historically is to use archival scoring of written records. The concept of integrative complexity (e.g., [15]) comes very close to AOT. The scoring system is based on two measures: differentiation and integration. Differentiation is the acknowledgment of multiple views or perspectives. It is essentially equivalent to the idea of fairness in AOT. (This is no coincidence. The concept of AOT has many historical antecedents, and integrative complexity is one of them.) Integration involves some sort of synthesis of the resulting views. It is more difficult to score. And, in fact, differentiation alone does much of the work in accounting for correlations between integrative complexity and other measures. Most studies of integrative complexity involve highly specialized samples of material, such as speeches made by legislators. Of possible relevance here, Thoemmes and Conway [51] found essentially no historical change in the complexity of speeches of U.S. presidents from George Washington to George W. Bush, but they did find several possible determinants other than historical time.

Now it is time for a guess. Recently, Emlen Metz, in her PhD dissertation (summarized in [45]) developed measures of AOT for middle-school children (roughly ages 12–13) in the U.S. The schools included private schools for the well-off and public schools in poor neighborhoods, but, it might be said, none of the “Christian” schools in the South, known for their emphasis on strict doctrine and opposition to some of the conclusions of modern science. What surprised everyone about the results of these tests, modeled after the adult versions but also including many items with open-ended answers, was that most students were near the ceiling. The expected correlations with other measures—highly significant because of the very large sample size—were low because of the low variance in all measure of AOT. Some of the open-ended items required students to put themselves in the position of someone holding a position now thought to be untenable, such as the claim that women should not be allowed to vote. Most students were able to do this, even though they disagreed with the conclusion. They had no particular resistance to trying to think of “arguments on the other side” from their own. They also dealt reasonably with hypothetical scenarios involving substantive arguments with peers.

Unfortunately, it was impossible to find historical data even on items taken from tests that have been used extensively over the last few decades. But it seemed likely to me that schools—public, private, rich, and poor—have adopted a norm of respect for others, and this norm has implications for how to deal with people who hold different views. This norm is part of AOT but not all of it. For example, it has little to do with (and may not generalize to) thought processes about individual decisions that do not involve conflicts of opinion, such as those concerning health or how to fix a toilet, and little to do with appropriate confidence.

I also suspect that culture in some parts of the world, and of the U.S., has moved farther from the sort of ideology that encourages AOT. Such “negative” cultural changes may result from the spread of fundamentalist or doctrinaire religions of many sorts (Muslim, Christian, Jewish, and Hindu), even in countries that have in the past embraced liberal, tolerant, doctrines (e.g., Turkey, Indonesia). It is as if more people want to declare their identity through membership in a religious group that regards other doctrines as potentially subversive and in need of suppression. Such cultural defenses may tend to spread, even to the point of discouraging children’s natural curiosity about outsiders.

Such cultural attitudes are part of the world’s problems. We see this specifically in religious opposition to polio vaccination in Nigeria and Pakistan, in one-issue voting on abortion in the U.S., and in the persistent Hindu/Muslim conflict in South Asia. But the same general attitudes prevent citizens from thinking well about other issues, and, more importantly, distort their evaluation of potential leaders and trusted authorities toward those who are most doctrinaire, those with the most unwarranted confidence.

## 5. Promoting AOT in Schools

The argument for AOT is thus simple. Errors of judgment, and poor decisions, are common. Especially when judgments of different people conflict, as in beliefs about religion or public policy, at least one of the parties must be incorrect in some way. How can we protect ourselves against such errors? The answer, the essence of AOT, was provided by J. S. Mill in the quote above. And it applies in spades when we judge our leaders.

What is needed is cultural change (or prevention of such change in the wrong direction), perhaps beginning with the norms of the classroom, but also in the news media, in political discourse, and in everyday norms of discussion. Even in classrooms that emphasize respect for the views of others, more is needed. In particular, people must understand the justification of AOT, just as I have sketched it in here. They must learn the basic relation between fair reflection and justified confidence. And they must come to understand how potential leaders and authorities can be evaluated in these terms. Understanding of AOT is not like understanding rocket science. It is more the basic physics of mass, weight, volume, and density (as assessed in “Volume and heaviness” test based on Piaget’s discoveries; as described by [44]).

Attempts to teach students, and others, to think better have been going on for some time, perhaps since the Renaissance [36]. Some recent efforts have been designed with the idea of increasing intelligence in mind, such as the “Venezuela project” [52]. Inspired by the writing and political activity of Luis Alberto Machado [53], the aim of this project was to increase intelligence by teaching thinking. An initial experiment in a large number of 7th grade classes was successful in raising IQ scores. But experiment did not lead to lasting changes in Venezuela’s political system. A change of government led to the closing of the “Ministry of Intelligence” (headed by Machado), the source of its support within the government. And the Ministry of Education was not equipped to extend the program to new schools by further developing the materials.^9^ Some time after the project closed, I attended a small meeting at Yale to discuss other ways of funding similar efforts elsewhere. (Japan and Saudi Arabia were mentioned as possibly interested. If only ...).

But it is not clear to me that this project could have provided an adequate basis for further development. An examination of the curriculum [54] suggests that the approach of this project was to “think of everything that might help and put it all together”. This was a perfectly reasonable approach given the primary goal, which was to try to show that intelligence *could* be increased through an educational intervention. Many of the lessons concerned specific subject matter, such as language, mathematics, solving common types of problems, or creative writing. Very little was specifically directed at highly general dispositions such as those I have discussed, although it seems quite likely that many of these dispositions were affected, if only as side effects of instruction in specific tasks.

I think that what is needed now is a method of teaching thinking that is based on a clear and simple theory that teachers can understand, such as the AOT theory I have sketched here (or something similar). A few smaller experiments have tested such ideas. Selz [55] described a few relevant studies in which he directly tried to increase intelligence by training in problem solving. In one, an experimental and a control group of students, aged 11 to 13, were given an intelligence test consisting of completion problems (stories with words left out); word-ordering problems (arranging words into a sentence); verbal-analogy problems; and number-series completions. The experimental group was given training on only the completion problems for 1 h on each of two successive days. The training was designed to make students take into account the requirements of the task, checking each possible answer to see if these requirements were met. Subjects were taught both to explain why answers did not meet the requirements and to justify answers when they seemed to fit. After the training, a second intelligence test was given. The experimental group showed substantial improvement not only on the completion test, on which they were trained, but also on all of the others, to roughly the same extent. Apparently, the students learned to be more critical of initial possibilities, seeking evidence against them as well as looking for alternative possibilities.

Perkins et al. [17] taught high school students to think in an actively open-minded way through a sixteen-session course that emphasized searching thoroughly for arguments on both sides of an issue. Students were taught that the arguments they consider when thinking about a controversial issue should be true (to the best of the thinker’s knowledge), relevant to the issue, and complete—that is, all important relevant arguments should be considered. Controversial issues were discussed in class, and students were explicitly encouraged to generate and evaluate (for truth and relevance) arguments on both sides, especially the other side. When students were asked to list arguments, the course nearly doubled the number of arguments that students gave on the other side from their own.

Gaskins and Baron conducted an 8-month training study in her school for reading-disabled children, the Benchmark School [22]. The teachers in the school (including Gaskins) identified three cognitive styles that they felt were holding many children back from academic success, even when their initial reading problems had been largely corrected. We called these styles *impulsiveness, rigidity*, and *nonpersistence*. *Impulsiveness* consisted of failing to think sufficiently on an individual problem or when answering a question. *Rigidity* consisted of an unwillingness to consider alternatives to an initial possibility concerning how something should be done or about the truth of some issue. *Nonpersistence* was the failure to complete extended activities, such as seat-work assignments; it can be taken as a sign of lack of motivation. Our training program was designed to overcome these biases by emphasizing three slogans: “Take time to think”; “Consider alternatives”; and “Keep at it.” The program was a success, according to teacher ratings of the styles we tried to train; the experimental group improved considerably and the control group hardly at all. The training also (weakly) affected ratings of academic performance given by teachers of children who had graduated from the Benchmark School and gone to other schools. In addition, children did slow down and take more time to think in a few different laboratory measures using tasks other than those used in training, including syllogisms and arithmetic word problems.

The Gaskins/Baron program was directed at both AOT and R/I, but the two others were directed at AOT. This is no coincidence, because their existence influences the development of the theory I sketched earlier. In sum, it seems that we can successfully teach AOT by encouraging it directly, and explaining to students why it helps.

To some extent, it could be argued that most people would learn how to think well on their own, but for the barriers set up by cultures that survive because they discourage questioning. Curiosity, for example, seems to be a common feature of childhood, yet parents and teachers often discourage it by telling children that certain questions should not be asked.

## 6. Conclusions

The Flynn effect may result from a kind of g factor of *influences* on IQ, including improvements in economic standard of living, public health, environmental quality, parental investment in children’s education, and so on, all of which influence each other and indeed are also influenced by improvements in the entire battery of abilities and dispositions that constitute IQ (along the lines suggested by [41]).

AOT, as one of many components of IQ, may differ from the rest. Low acceptance of AOT is compatible with cultures that do well by the other criteria. (Saudi Arabia comes to mind.) Education can be economically useful without encouraging AOT. Thus, AOT will be subject to other influences, particularly the ebb and flow of the worldwide “culture wars”. In those wars, it is on the side of the Enlightenment, and it is opposed by various traditions that emphasize the role of “the heart”, “faith”, “intuition” and (unquestioned) “authority”. If we had been paying attention, we probably would have seen both increases and decreases in AOT in different social environments over the last century.

If AOT is part of a culture war, should its advocates be actively open-minded about the other side? Should we try to be “balanced” in our discussion of alternative ways of coming to have beliefs? There are many manifestations of this question, e.g., the controversy about whether reflective classroom discussion about the theory of evolution must give some time, or equal time, to creationism. Does equal time amount to “false balance”?

My answer is that the most important thing is to teach people to *understand* the arguments about why, and when, AOT is superior to other forms of reasoning. They must understand it as a “design” in Perkins’ [56] sense. That is, they must know its purposes (coming up with the best answer, with appropriate confidence), its structure (testing, and revising or replacing, tentative conclusions, maintaining appropriate confidence, and so on) and the arguments about why this structure serves the purposes. Students can understand something without accepting it, so this is not indoctrination in its pure form. Of course, we do know what the outcome will be: if we teach understanding of some concept and test this understanding (as we must do, if we want to teach it effectively), then in fact more students will accept it. But we are applying our incentives to understanding, and acceptance is a beneficial side effect.

If the culture warriors from the other side challenge us, then we must argue with them respectfully, but firmly. Is AOT special in this way? Does instruction in physics and astronomy affect how people think about the cosmos, in ways that might conflict with religious doctrine? And, of course, we must ask why we should accept someone’s conclusions if all the arguments for them come from their intuition or from historical longevity.

The benefits of increased AOT may show up in economic progress, but they will have special effects on politics. Citizens do not need to be very “smart” in the usual sense to know when they do not know something, and to figure out which authorities are trustworthy, by understanding what those authorities have done, or not done, to reach their conclusions. As I learned in college when I switched my major to psychology, psychology is easier than real science.

## Footnotes

1. It would be convenient for my argument if China did not exist. It is certainly not democratic, yet it is making more progress on most fronts than many well-established democracies. This may be less of a contradiction than it seems. The government pays attention to signals from the people, even if those are in the form of opinion polls rather than voting, and the ruling Communist party is quite large and is somewhat sensitive to the concerns of its members. The government has thus moved to reduce corruption and strengthen the rule of law. In sum, Chinese citizens do have some influence.

2. Cosmopolitanism is a continuum of breadth of political concern, one end of which is a concern for all humanity (or all sentient life) now and in the future. Anti-moralism is opposition to the imposition of moral principles on others when the principles themselves depend on particular commitments of faith that cannot be defended to those who are affected.

3. It would digress too far to deal with the potential circularity of “rational goals” in the definition of “rational thinking”. In brief, we can define a subset of rational thinking without this limitation, then discuss the rationality of goals, and so on.

4. Similarly, a parallel literature on “critical thinking” (e.g., [15]) seems concerned with the full range of skills, some quite specific to certain content, that make for effectiveness in what is understood to be good thinking. Many writers in this tradition take the term “critical” almost literally, so that their concern is to encourage skepticism. Yet, AOT also gives us reasons to trust, as well as to find fault.

5. The direction parameter is not always under the thinker’s control. Search of external data sometimes prevents it, and many types of inferences, such as arithmetic calculation, are fixed and cannot be modified.

6. In the first edition of *Thinking and deciding* (1988), I used the term “myside bias” (which had been used previously by David Perkins) because “confirmation bias” was being used for something that was not a bias. This usage seems to have stopped. Now it seems that we have two terms for the same idea.

7. The idea that R/I can be assessed from Raven’s test and is part of g is not a radical one. Arthur Jensen [48], a strong defender of the traditional approach to the g factor, writes (pp. 617–619): “Reflective persons tend to delay responses in answering test items involving initial uncertainty, and as a result their performance gains in accuracy. The payoff of a reflective attitude is greatest in tasks that call for careful analysis of possible response alternatives, as is characteristic, for example, of multiple-choice nonverbal tests such as the Raven’s Matrices.” After noting the correlation between R/I and IQ, he suggests “that much of the correlation may be due to an intrinsic relationship between g and reflectivity, that is, one of the manifestations of g ability is reflectiveness.”

8. In principle, thinking could be systematically biased against currently favored possibilities, but I know of no results showing that a general dimension of direction can go “below 0” in this way.

9. Personal communication from Prof. Jorge Dominguez (Harvard), who helped design the program and has followed education in Venezuela over the years.
